# Anti-Psl Targeting of *Pseudomonas aeruginosa* Biofilms for Neutrophil-Mediated Disruption

**DOI:** 10.1038/s41598-017-16215-6

**Published:** 2017-11-22

**Authors:** Valerie A. Ray, Preston J. Hill, C. Kendall Stover, Sashwati Roy, Chandan K. Sen, Li Yu, Daniel J. Wozniak, Antonio DiGiandomenico

**Affiliations:** 1 Center for Microbial Interface Biology, Departments of Microbial Infection and Immunity, Microbiology, Ohio State University, Columbus, OH 43210 USA; 2 Department of Infectious Diseases, MedImmune, LLC, Gaithersburg, MD 20878 USA; 3 Comprehensive Wound Center, Davis Heart and Lung Research Institute, Center for Regenerative Medicine and Cell Based Therapies, Ohio State University Medical Center, Columbus, OH 43210 USA; 4 Department of Surgery, College of Veterinary Medicine, Ohio State University, Columbus, OH 43210 USA; 5 Translational Sciences, MedImmune, LLC, Gaithersburg, MD 20878 USA

## Abstract

Bacterial biofilms are recalcitrant to antibiotic therapy and a major cause of persistent and recurrent infections. New antibody-based therapies may offer potential to target biofilm specific components for host-cell mediated bacterial clearance. For *Pseudomonas aeruginosa*, human monoclonal antibodies (mAbs) targeting the Psl biofilm exopolysaccharide exhibit protective activity against planktonic bacteria in acute infection models. However, anti-Psl mAb activity against *P*. *aeruginosa* biofilms is unknown. Here, we demonstrate that anti-Psl mAbs targeting three distinct Psl epitopes exhibit stratified binding in mature *in vitro* biofilms and bind Psl within the context of a chronic biofilm infection. These mAbs also exhibit differential abilities to inhibit early biofilm events and reduce biomass from mature biofilms in the presence of neutrophils. Importantly, a mAb mixture with neutrophils exhibited the greatest biomass reduction, which was further enhanced when combined with meropenem, a common anti-Pseudomonal carbapenem antibiotic. Moreover, neutrophil-mediated killing of biofilm bacteria correlated with the evident mAb epitope stratification within the biofilm. Overall, our results suggest that anti-Psl mAbs might be promising candidates for adjunctive use with antibiotics to inhibit/disrupt *P*. *aeruginosa* biofilms as a result of chronic infection.

## Introduction

Many chronic infections (~60%), including otitis media^1^, keratitis^2^, CF airway^3,4^, burns^5,6^, wounds^7,8^, and surgical sites^9^ are due to biofilms^10,11^. While in this lifestyle, bacteria produce and secrete an extracellular matrix composed of polysaccharides, proteins, and extracellular DNA (eDNA) that encase and shield the bacteria against chemotherapeutic and host assaults^12,13^. Therefore, much effort has been directed at developing novel treatments for biofilm infections since they are largely recalcitrant to standard therapeutics.

One clinically relevant organism that causes a variety of the aforementioned infections is *Pseudomonas aeruginosa*^14^. With regards to biofilm formation, *P*. *aeruginosa* is considered a model organism^15^. One key component of the *P*. *aeruginosa* biofilm matrix is the polysaccharide Psl, which is produced by proteins encoded within the *p*olysaccharide *s*ynthesis *l*ocus^16–18^. Psl is both cell-free and surface-associated^19,20^. The structure of cell-free Psl is composed of a repeating pentasaccharide of D-mannose, L-rhamnose, and D-glucose^19^ (Supplementary Fig. S1A). Since Psl serves both a structural^21–23^ and protective^24–27^ function during biofilm formation, it may be an ideal target for novel therapeutic options. Human monoclonal antibodies (mAbs) targeting three distinct epitopes within Psl, referred to as class I, II and III, were recently described^28^. To identify anti-Psl mAb epitope binding requirements, we constructed a panel of synthetic Psl oligosaccharides followed by evaluation of anti-Psl mAb binding to each (Supplementary Fig. S1B–E)^29^. The class II epitope binding mAb, WapR001, recognized all oligosaccharide derivatives, while the class III epitope binding mAb, WapR016, recognized the hexasaccharide containing a terminal glucose residue and weakly bound to a decasaccharide (comprised of two Psl pentasaccharide units) (Supplementary Fig. S1B)^29^. Unexpectedly, mAbs that bound the class I epitope (Cam003 or its affinity optimized derivative, Psl0096), which were also the most active in promoting opsonophagocytic killing (OPK) or in preventing *P*. *aeruginosa* binding to epithelial cells^28^, did not bind any of the synthetized oligosaccharides. Interestingly, this epitope was associated with an acyl chain modification that is sensitive to mild alkaline exposure^29^.

Although each anti-Psl mAb exhibits functional *in vitro* activity (OPK and anti-cell attachment activity) against planktonic *P*. *aeruginosa* and are protective in acute murine infection models^28^, no studies have examined whether they can recognize Psl within *P*. *aeruginosa* biofilms, and if so, whether they are capable of promoting biofilm clearance alone or in the presence of innate immune effector cells. Indeed, the potential impact of antibodies on biofilm formation or disruption has not been adequately studied in a wider range of pathogen systems. Using a porcine thermal injury model in which injured skin is chronically infected with *P*. *aeruginosa*, we demonstrate that all three Psl epitopes are recognized by anti-Psl mAbs. We show that anti-Psl mAbs exhibit differential abilities to block early biofilm events, such as surface attachment and bacterial auto-aggregation. In addition, all anti-Psl mAbs can serve as opsonins and promote biofilm biomass reduction in the presence of human neutrophils, a function greatly enhanced when combined with meropenem. Furthermore, we show that the class I, II and III Psl epitopes are stratified within mature biofilms, resulting in a complex staining pattern that correlated with the ability of neutrophils to access and kill bacteria within the biofilm.

## Results

### Anti-Psl mAbs differentially stain *P*. *aeruginosa* biofilms

**Figure 1:**
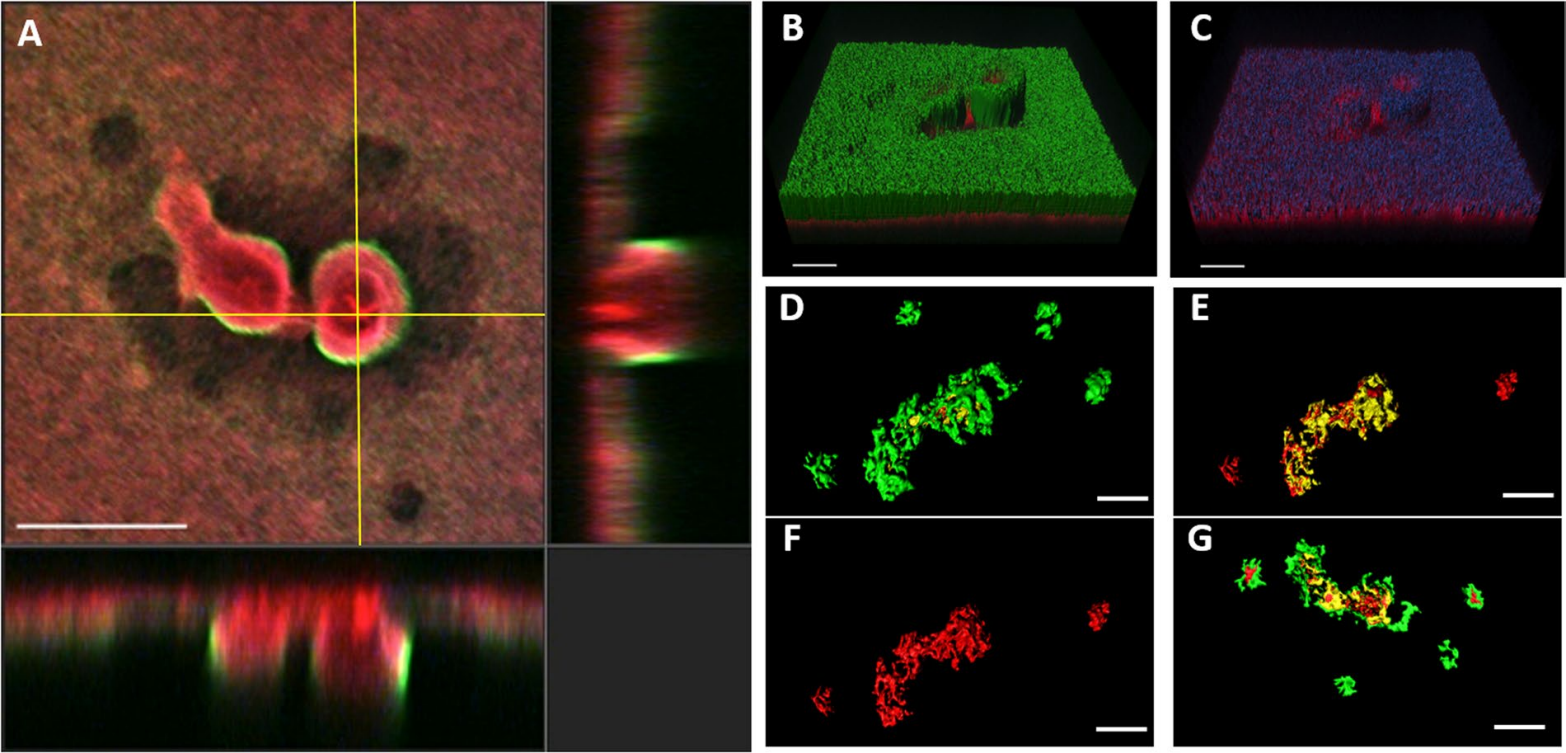
Anti-Psl antibodies differentially stain PAO1 biofilms. (**A**–**C**) Staining of flow (18h) and (**D**–**G**) static grown (48 h) biofilms with anti-Psl mAbs: class I (green), class II (blue **A**–**C** or yellow **D**–**G**), and class III (red). (**A**) CLSM image at 10x magnification and (**B**,**C**) IMARIS processed image with (**B**) all three layers or (C) class I (green) layer removed. (**D**–**G**) IMARIS processed static biofilms at 100x magnification from (**D**) top, (**E**) class I (green) layer removed, (**F**) class I (green) and class II (yellow) layers removed, and (**G**) bottom. Scale bars (**A**) 100 µm, (**B**,**C**) 40 µm, and (**D**–**G**) 150 µm.

**Figure 2:**
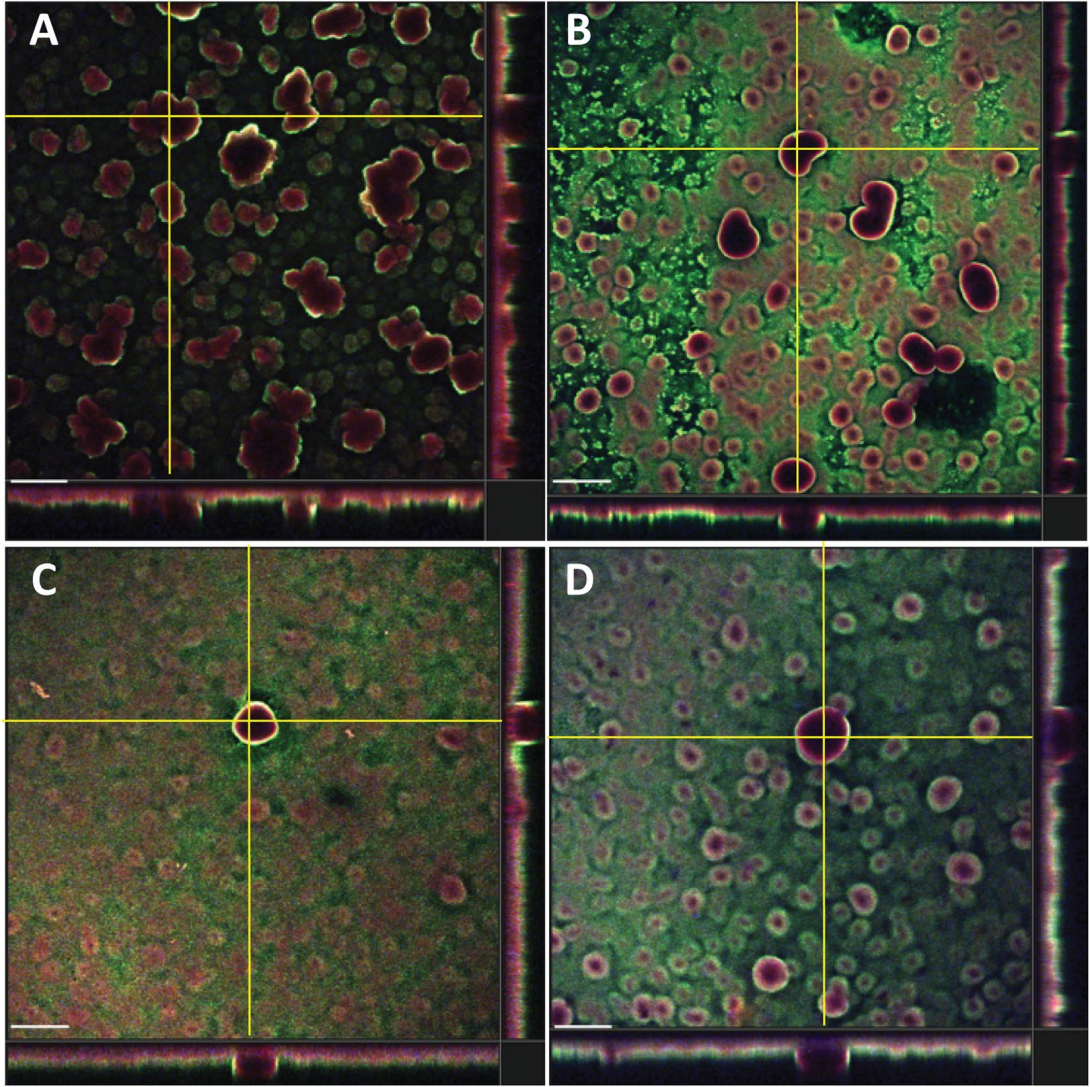
Anti-Psl mAbs exhibit differential staining of biofilms from several *P*. *aeruginosa* isolates. Flow cell grown biofilms were stained with the anti-Psl mAbs (class I – green, class II – blue, and class III – red) and imaged via CLSM at 10x magnification. Images are of a section from a z-stack to depict staining pattern. (**A**) Cystic fibrosis isolate CF127^52^. (**B**) XDR strain ARC3928^36^. (**C**) Environmental isolate MSH3^52^. (**D**) Corneal isolate 6077^52^. Scale bars represent 150 µm.

Since the anti-Psl mAbs recognize unique epitopes of Psl on planktonic *P*. *aeruginosa* (class I, class II, and class III;^29^), we sought to evaluate whether these epitopes were expressed within mature biofilms. To do this, we grew mature PAO1 biofilms under either flow (18 hour biofilms) or static conditions (24 hour biofilms) followed by staining and analysis via confocal laser scanning microscopy (CLSM) using fluorescently labeled versions of each anti-Psl mAb. Intriguingly, a combination of these mAbs resulted in a differential staining pattern, such that the class I mAb stained primarily at the top of the biofilm, whereas the class II and III mAbs stained primarily beneath this layer. The class III mAb primarily stained the base of the biofilm with the class II mAb mostly staining between the class I and III mAbs (Fig. 1 and Supplementary Movie S1). Each individual mAb also stained the biofilm when used alone (Supplementary Fig. S2). No differences in the staining pattern was observed between biofilms grown in flow cells up to 18 hours (Fig. 1A–C) or under static conditions up to 48 hours (Fig. 1D,E; data not shown) or when interchanging the fluorophores on the mAbs (data not shown). No signal was observed when staining biofilms with a labelled control IgG (data not shown). In addition, the staining pattern was conserved in other laboratory and clinical isolates of *P*. *aeruginosa* tested (Fig. 2A–D), indicating that this is not a strain-specific phenomenon. Overall, these data suggest a complex organization of Psl within mature *in vitro* biofilms.

### All three Psl epitopes are expressed and recognized *in vivo*

**Figure 3:**
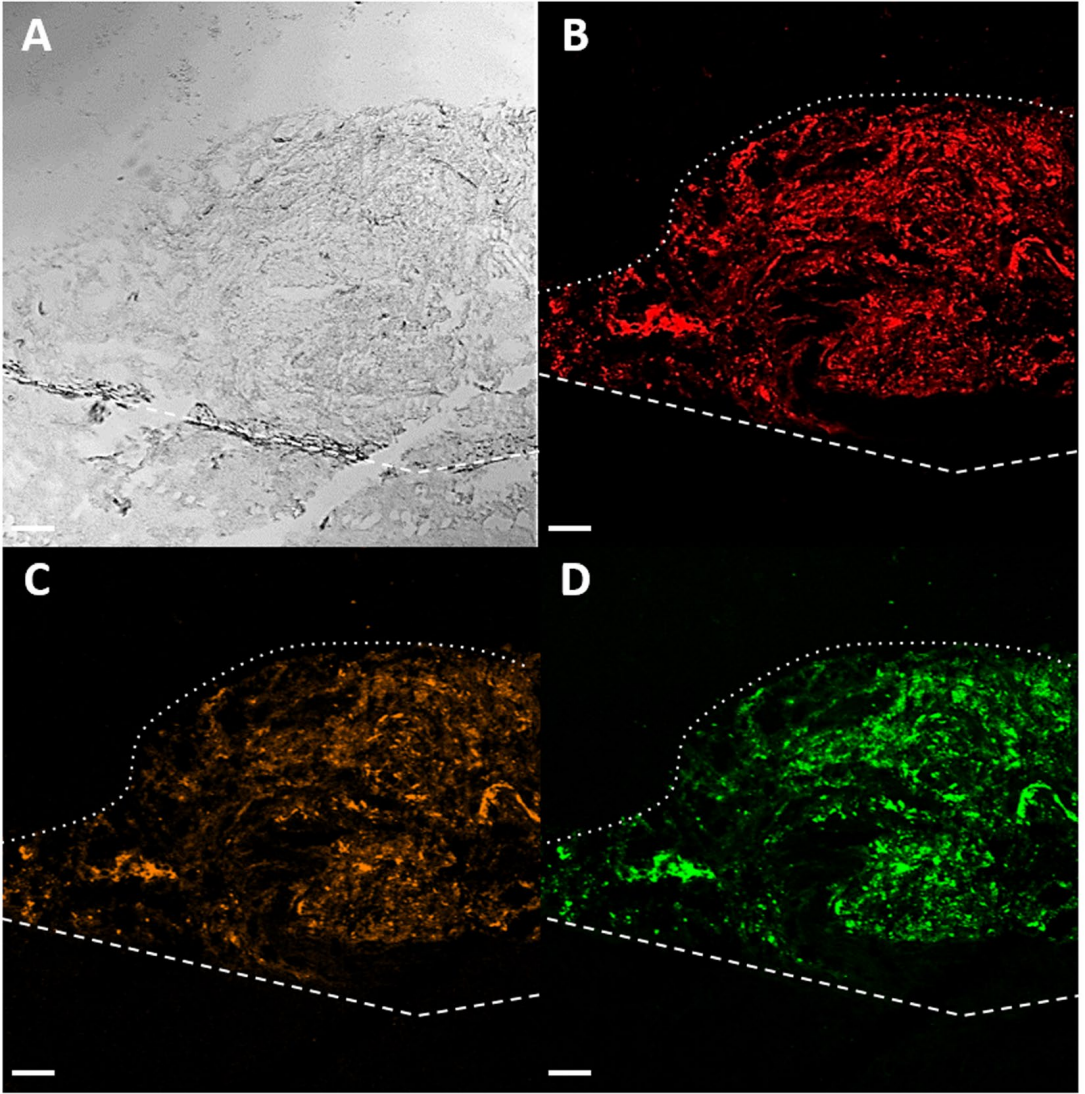
Anti-Psl antibodies recognize Psl in a chronic *P*. *aeruginosa* infection. *P*. *aeruginosa* infected skin from thermally injured pigs was stained with fluorescently labeled anti-Psl mAbs. (**A**) DIC image, (**B**) class I anti-Psl mAb (red), (**C**) class II anti-Psl mAb (orange), and (**D**) class III anti-Psl mAb (green). The wound bed is below the white dotted line and white dashed line represents below the wound bed. Scale bars represent 100 µm.

Biofilm formation is a trait often observed in persistent/chronic bacterial infections. Given the important role of Psl in *P*. *aeruginosa* biofilm formation and maintenance^19,21,30–33^, we sought to evaluate whether the anti-Psl mAbs could recognize Psl in chronically infected tissue. For these experiments, we used skin from thermally injured pigs infected with *P*. *aeruginosa* strain PAO1 since this model had previously been shown to closely mimic human wound infections^6^. Skin was harvested from animals fourteen days post-infection followed by staining with fluorescently labelled anti-Psl mAbs. We demonstrate that mAbs targeting all three Psl epitopes reacted with infected tissue (Fig. 3), while no reactivity was observed using a fluorescently labeled isotype control IgG (Supplementary Fig. S3). Furthermore, non-reactivity of labelled anti-Psl mAbs was observed when using non-infected thermally injured tissue, confirming the specificity of the mAbs (data not shown). These results indicate that all three Psl epitopes are expressed and accessible by mAbs in chronically infected tissue.

### Anti-Psl mAbs decrease attachment to abiotic and biotic surfaces

**Figure 4:**
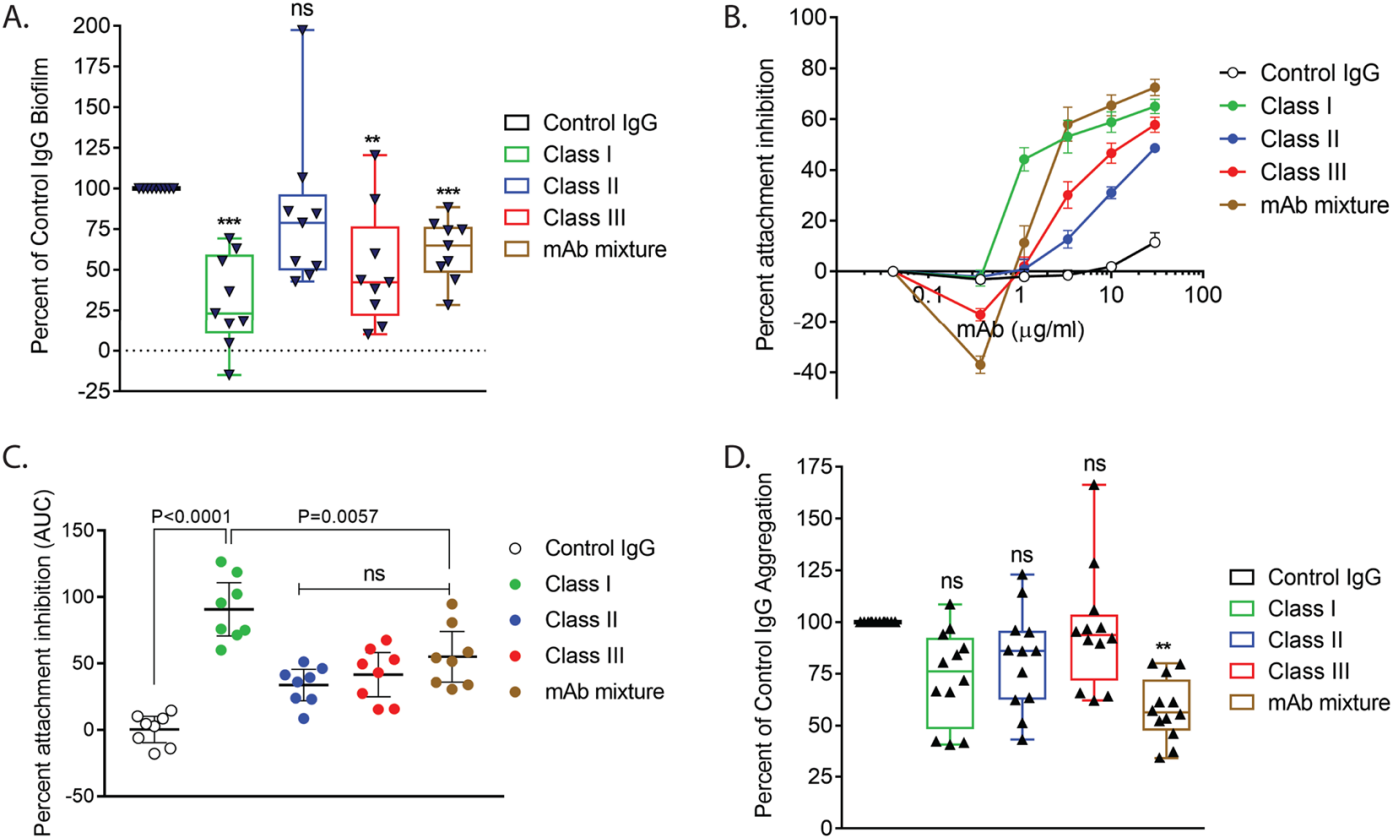
Anti-Psl mAbs reduce attachment and cell-cell aggregation. (**A**) Anti-Psl mAbs (10 µg/ml) inhibit *P*. *aeruginosa* attachment to an abiotic surface. ***p < 0.0001, **p = 0.0025, ***p = 0.036. (**B**) Anti-Psl mAbs inhibit attachment to epithelial cells. (**C**) Epithelial cell attachment assay calculated area under the curve (AUC). (**D**) Aggregation in liquid culture. **p < 0.0028. (**A**,**D**) statistics were determined using Student’s t-test, n = 3 or more for all experiments. (**C**) The AUC was estimated using the Trapezoidal rule with group comparisons analyzed by one-way ANOVA with the Dunnett’s multiple comparison test.

To understand whether each anti-Psl mAb impacts the ability of *P*. *aeruginosa* to initiate biofilms, we first examined the ability of each mAb to affect Psl-mediated bacterial attachment. Psl is a key factor in attachment to both biotic and abiotic surfaces^19,21,30–33^. While previous studies have shown that each anti-Psl mAb can prevent attachment of *P*. *aeruginosa* to cultured epithelial cells^28^, it is unclear whether these mAbs can block Psl-mediated attachment to an abiotic surface. Therefore, we examined the ability of *P*. *aeruginosa* strain PAO1 to bind to an abiotic surface in the presence of the anti-Psl mAbs (individually or in combination) as compared to an isotype control IgG. We found that mAbs targeting the class I or III epitopes reduced Psl-mediated attachment by ~70 and ~50%, respectively, while treatment with a class II epitope targeting mAb did not significantly reduce attachment (Fig. 4A). Inhibition of attachment was dose-dependent as activity was reduced with decreasing concentrations of each mAb (data not shown). Interestingly, these results mirrored the activity of each mAb in preventing *P*. *aeruginosa* attachment to an epithelial cell line (class I > class III > class II) (Fig. 4B). In this latter experiment, inhibition of attachment was dose-dependent with activity waning at lower antibody dilutions (Fig. 4B). Statistical significance was determined by calculating the area under the curve, with the anti-Psl class I mAb identified as the most active mAb in preventing *P*. *aeruginosa* attachment to epithelial cells (Fig. 4C). We also observed a reduction in attachment when a combination of all three mAbs was used (mAb mixture), albeit to a lesser degree when compared to the anti-Psl class I epitope mAb alone for both abiotic and biotic surfaces (Fig. 4A–C). This latter result was not surprising, however, since mAbs binding the class I and III epitopes were shown to partially compete for epitope binding against planktonic *P*. *aeruginosa*^28^, which could potentially explain the reduced activity of the anti-Psl mAb mixture. Similar results were observed when evaluating mAb activity against additional *P*. *aeruginosa* strains (data not shown). Overall, this data suggests mAbs targeting the anti-Psl class I and III epitopes, individually or in combination, reduce attachment of *P*. *aeruginosa* to both abiotic and biotic surfaces, thus hindering the ability of the bacterium to form a biofilm.

### Anti-Psl mAbs inhibit cell-cell aggregation

Psl is not only involved in bacteria-surface contacts, but also cell-cell interactions, another important biofilm property^17,34,35^. To address whether these antibodies could prevent early aggregation events, we utilized a version of PAO1 that overproduces Psl (WFPA801;^21^); as a negative and normalization control, we used an aggregation and Psl-defective strain of PAO1 (PAO1*∆psl*). Although we observed a trend in reducing cell-cell aggregation by individual anti-Psl mAbs, the decreases were not significant when compared to the control IgG (Fig. 4D). However, we did observe a significant reduction when all three anti-Psl mAbs were added in combination (Fig. 4D). These results suggest that in addition to blocking *P*. *aeruginosa* attachment to biotic and abiotic surfaces, mAb targeting of Psl may also inhibit bacterial aggregation, thus reducing Psl-mediated cell-cell interactions.

### Anti-Psl mAbs mediate phagocytic activity against mature biofilms

**Figure 5:**
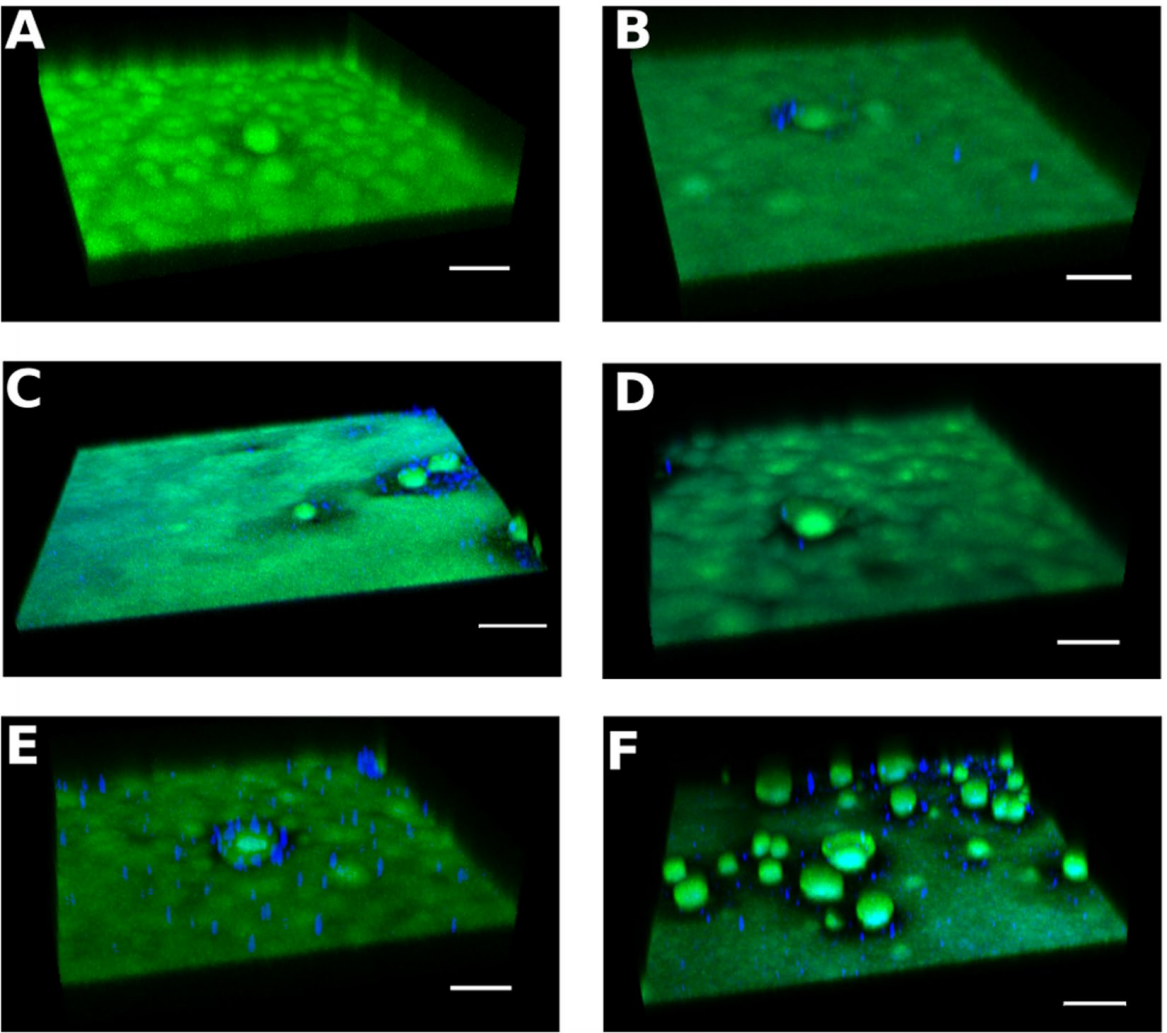
Anti-Psl mAbs promote biofilm biomass reduction in the presence of neutrophils. Mature flow grown biofilms of PAO1 (green) were incubated with either (**A**) anti-Psl mAb alone (20 µg/ml) or mAb mixture (6.7 µg/ml ea.), shown as a representative image or (**B**–**F**) in the presence of neutrophils (blue): (**B**) IgG control mAb (20 µg/ml), (**C**) class I mAb, (**D**) class II mAb, (**E**) class III mAb, or (**F**) a combination of all three mAbs. Scale bars represent 100 µM.

In addition to inhibiting *P*. *aeruginosa* attachment to biotic^28^ and abiotic surfaces (Fig. 4A,B), all three anti-Psl mAbs mediate opsonophagocytic killing (OPK) of planktonic *P*. *aeruginosa*^28^. The mAb targeting the class I epitope exhibits enhanced OPK activity compared to mAbs targeting the class II and III Psl epitopes (class I > class III > class II)^28^. Given these results, we next identified whether anti-Psl mAbs could disrupt mature biofilms. In the absence of effector cells (primary human-derived neutrophils), individual anti-Psl mAbs (20 µg/ml) or a mAb mixture (6.7 μg/ml for each mAb) were unable to promote disruption of the biofilm (Fig. 5A). However, in the presence of primary human neutrophils, we observed a reduction in biofilm density in comparison to control IgG of 36.2 (p < 0.001), 20.3 (p < 0.05), and 13.3% for individual class I, class III, or class II anti-Psl mAbs, respectively (Fig. 5C–E; Table 1); no reduction was observed with an isotype control or non-treated biofilms under these conditions (Fig. 5B and data not shown). When all three mAbs were combined, biomass reduction was increased (p < 0.0001 vs control IgG) (Fig. 5F; Table 1). To confirm anti-Psl mAb activity against mature biofilms, we evaluated activity against several recent *P*. *aeruginosa* clinical isolates, including an extensively drug resistant (XDR) strain, ARC3928, which is resistant to all known anti-Pseudomonal antibiotics with the exception of colistin^36^. The activity of individual mAbs against strain CF127 was similar as we observed against PAO1 (Table 1). The greatest reduction in biofilm density was again observed in the presence of the class I anti-Psl mAb and the mAb mixture (Table 1). For perspective, a peak serum concentration of 5 μg/ml in a 20 g mouse is typically achieved with a mAb dosage of 1.0 mg/kg. While a similar trend in biomass reduction was observed for all anti-Psl mAbs and the mAb mixture against XDR strain ARC3928, statistical significance vs. control IgG was not achieved (Table 1). We propose that the reduction in biomass observed with the anti-Psl mAb mixture was likely due to neutrophil access rather than activation, since the individual anti-Psl mAbs, as well as the combination, promoted an oxidative burst response from neutrophils when incubated with planktonic *P*. *aeruginosa* (Supplementary Fig. S4). Overall, these data suggest that combinations of anti-Psl mAbs are capable of promoting clearance of *P*. *aeruginosa* biofilms in the presence of primary human neutrophils.

**Table 1:** *P*. *aeruginosa* biomass reduction after primary neutrophil and mAb treatment.

Strain	Condition	% Biomass Reduction^a^	p-Value^b^
PAO1	Class I	36.2+/−5.3	<0.001
	Class II	13.3+/−9.0	n.s.
	Class III	20.3+/−6.4	<0.05
	mAb mixture	43.4+/−5.1	<0.0001
CF127	Class I	28.7+/−4.6	<0.05
	Class II	6.6+/−10.2	n.s.
	Class III	18.7+/−8.2	n.s.
	mAb mixture	39.0+/−8.0	<0.05
ARC3929	Class I	19.2+/−7.5	n.s.
	Class II	14.5+/−4.9	n.s.
	Class III	14.4+/−6.9	n.s.
	mAb mixture	30.8+/−9.9	n.s.

^a^Compared to IgG control treated biofilms +/−SEM.

^b^n.s. means not significant with p-Value > 0.05.

### Combination of meropenem and anti-Psl mAbs enhances biofilm clearance

**Figure 6:**
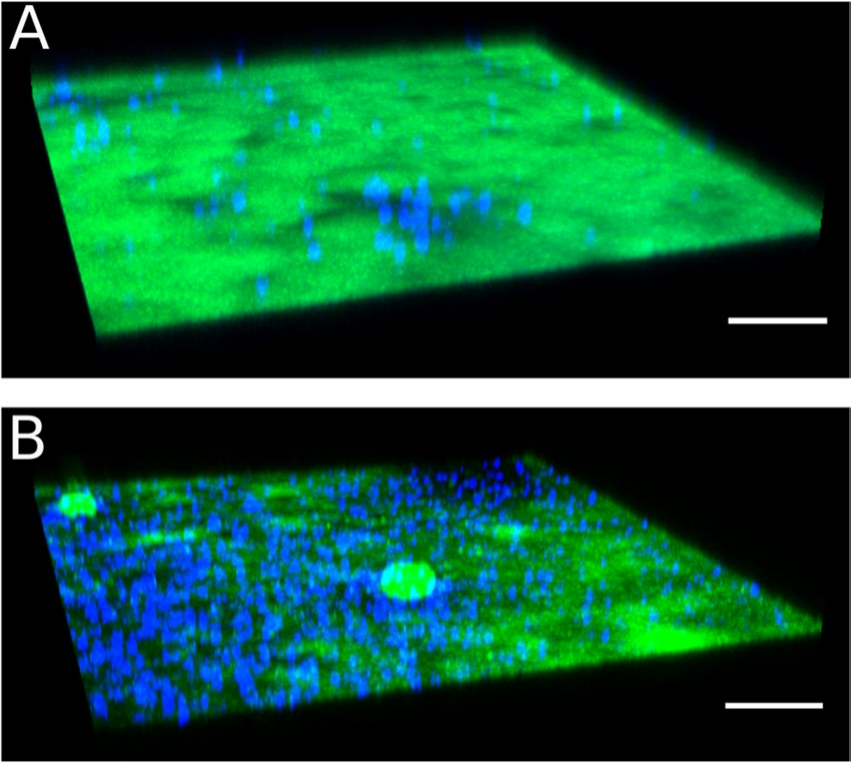
Biofilm disruption is enhanced following mAb and meropenem treatment. Mature flow grown biofilms of PAO1 (green) were incubated with neutrophils (blue) and (**A**) MEM alone (30 µg/ml) or (**B**) an anti-Psl mAb mixture (6.7 µg/ml ea.) and MEM (30 µg/ml). Scale bars represent 100 µm.

Since we observed enhanced biomass reduction with the combination of the three anti-Psl mAbs and neutrophils, we next asked whether this reduction could be further enhanced by the addition of meropenem (MEM), a common carbapenem antibiotic used to treat *P*. *aeruginosa* infections. The MIC of MEM for planktonic PAO1 is 2 μg/ml, while the minimum biofilm eradication concentration (MBEC) of MEM for this strain is approximately 1000 µg/ml^37^. For our studies, we utilized a range of physiologic MEM concentrations in these experiments to evaluate whether adjunctive mAb treatment would enhance biomass reduction. MEM concentrations of 0.5 and 3 μg/ml alone yielded limited reduction in biomass at 7.9% and 12.8%, respectively (Table 2). When combined with the anti-Psl mAb mixture, biofilm reduction was minimal, increasing to 52.9% and 60.3%, respectively (Fig. 6; Table 2). However, increasing MEM to 30 μg/ml, which is similar to exposure in human serum after 30 minute IV infusion of 500 mg (Cmax - 30.3 μg/ml)^38^ significantly increased biomass reduction (70.0%) when compared to MEM (30 µg/ml) (p < 0.05) alone or the mAb mixture alone (p < 0.01) (Table 2). Interestingly, the mAb mixture was more effective than MEM when either drug was used alone in reducing biofilm biomass at the tested concentrations. Overall, these data suggest that a combination of the anti-Psl mAbs and MEM, in the presence of primary neutrophils, enhance *in vitro* clearance of mature biofilms.

**Table 2:** *P*. *aeruginosa* biomass reduction after primary neutrophils, MEM, and mAb treatment.

	Condition	% Biomass Reduction^a^	p-Value^b^		
			vs. Control IgG	vs. mAb mixture	vs. MEM
mAb mixture	—	52.6 +/− 3.4	<0.001	—	—
MEM 0.5 µg/ml	−mAB	11.0 +/− 4.4	n.s.	<0.01	—
	+mAB	52.9 +/− 6.2	<0.01	n.s.	<0.0001
MEM 3 µg/ml	−mAB	11.2 +/− 5.1	n.s.	<0.01	—
	+mAB	60.3 +/− 5.9	<0.01	n.s.	<0.0001
MEM 30 µg/ml	−mAB	29.2 +/− 9.2	n.s.	n.s.	—
	+mAB	70.0 +/− 3.3	<0.001	<0.01	<0.05

^a^Compared to IgG control treated biofilms +/−SEM.

^b^n.s. means not significant with p-Value > 0.05.

## Discussion

*P*. *aeruginosa* infections are often refractory to antibiotic therapy due, in part, to the ability of this pathogen to form biofilms. The lack of antibiotic activity against biofilm embedded bacteria and the now widely accepted recognition of broad-spectrum antibiotic impact on the beneficial microbiota underscores the need for alternative approaches for treatment of serious *P*. *aeruginosa* disease. Pathogen-specific monoclonal antibodies (mAbs) against this organism have shown considerable potential in preclinical infection models but have not been evaluated for their impact on biofilms. Specifically, the identification of mAbs targeting the serotype-independent Psl exopolysaccharide, an important component of *P*. *aeruginosa* biofilms, were shown to mediate opsonophagocytic killing (OPK) and anti-cell attachment activity against planktonic *P*. *aeruginosa* while also exhibiting potent protective activity in multiple *P*. *aeruginosa* acute infection models^28^. Here we sought to determine whether anti-Psl mAbs targeting three unique epitopes were active in preventing *P*. *aeruginosa* biofilm formation and whether they exhibited activity in disrupting mature biofilms in the presence of host effector cells.

We first sought to establish whether mAbs could recognize Psl in chronically infected tissue. To perform this, we evaluated skin from thermally injured pigs that had been colonized with *P*. *aeruginosa* for fourteen days. Indeed, mAbs targeting all three epitopes strongly reacted with the infected tissue, confirming that Psl is a highly abundant target and is mAb accessible in a chronic infection (Fig. 3). This finding was expected given that these mAbs were identified from phage libraries derived from patients recovering from *P*. *aeruginosa* infections^28^. Interestingly, the identification of anti-Psl mAbs was rare even though phage libraries are known to be extraordinarily diverse^28^. More recent work confirmed the lack of robust immune response to Psl. Analysis of serum from individuals harboring confirmed *P*. *aeruginosa* bloodstream or respiratory tract infections, in which the infecting isolates were confirmed to express Psl *in vitro*, largely lacked significant Psl-specific antibody titers^39^. This result is in spite of > 90% of clinical isolates being capable of expressing Psl under *in vitro* conditions^28,36,39,40^. This finding is consistent with the role of Psl in shielding the bacterium from innate immune recognition^25,41^. These results highlight the potential therapeutic benefit of anti-Psl mAbs in mediating immune recognition and clearance of the bacterium, while potentially preventing systemic spread, for example, from a nidus of infection such as chronically infected burned tissue.

We also found that anti-Psl mAbs reduced early stages of biofilm formation, specifically attachment to surfaces and cell-cell aggregation, in an epitope dependent manner. The exact mechanism of inhibition is currently unclear, however our data suggests Psl-mediated attachment to either biotic or abiotic surfaces is similar, perhaps mediated by steric hindrance from mAb binding. Interestingly, mAbs targeting the class I and III epitopes significantly reduced abiotic cell attachment and inhibited aggregation while the mAb targeting the class II epitope was unable to perform either function efficiently (Fig. 4A). However, the class II epitope mAb exhibited relatively strong biotic anti-cell attachment activity, albeit below the activity observed for the class I and III epitope mAbs in this assay (Fig. 4B). One explanation for this overall observation is that at these early stages of biofilm formation, the epitope recognized by the Class II mAb might be partially occluded or mAb inaccessible. This might also be the case in mature biofilms, as we find that the class II epitope appears to localize between the class I and class III epitopes (Fig. 2).

We also observed that anti-Psl mAbs disrupted mature biofilms, from multiple *P*. *aeruginosa* strains, in the presence of primary human neutrophils. Similar to the anti-cell attachment activity assays, the class I and III epitope mAbs were the most active in promoting clearance or disruption of the biofilm, while a combination of the three mAbs at the same protein concentration (6.7 μg/ml each) led to overall enhanced reduction in biofilm density compared to the individual mAbs (20 μg/ml) (Fig. 5; Table 1). Interestingly, anti-Psl mAbs were more effective in mediating clearance than MEM alone, even though the *P*. *aeruginosa* strain (PAO1) used in these experiments are sensitive to MEM (MIC against planktonic bacteria - 2 μg/ml). These results are consistent with biofilms being notoriously recalcitrant to antibiotic treatment at physiologic reasonable concentrations. Importantly, adjunctive use of mAbs with MEM at 0.5 and 3 μg/ml resulted in additive reduction of biomass, when compared to using either drug alone (Fig. 6; Table 2).

Fluorescent mAb staining of biofilms generated under flow or static conditions revealed that each anti-Psl epitope binding apparently localized within different layers of the biofilm. The class I and III epitope mAbs primarily stained the surface and base of biofilms, respectively, while the class II epitope mAb was localized in between the class I and III regions (Fig. 2). The stratification of epitope accessibility correlated with the observed activity in the biofilm OPK assay, suggesting reduced killing observed with the class II and III epitope mAbs was likely due to neutrophil access rather than activation state. Interestingly, most of the disruption with the mAb mixture occurred on the biofilm mat rather than the tower structures. Since our staining results suggest that the class II epitope is indeed present in mature biofilms (Fig. 2), it seems unlikely that lower biofilm impact observed with this mAb is simply a matter of epitope occlusion, especially since the Class III mAb epitope appears near the bottom of the biofilm (Fig. 2) yet still promotes inhibition of early biofilm events (Fig. 4) and disruption of mature biofilms in the presence of neutrophils (Fig. 5).

The observed Psl epitope stratification is puzzling given that all three anti-Psl mAb classes are capable of binding planktonic bacteria^28^. Our data suggest that different Psl forms may either be involved in structuring of the biofilm or are simply a signature for the phenotypically distinct subpopulations within biofilms. A variety of explanations can potentially account for this observation. For example, the class I epitope could be produced by planktonic cells, which would presumably be found near the outside of the structure (aerobic environment), while the class II and III epitopes could be produced by cells further within the structure that are experiencing different microenvironments (i.e., decreased oxygen, change in nutrients, and/or increased waste). In addition, cells within the class II and III layers are likely metabolically less active, which might result in the observed epitope heterogeneity. While many other questions remain, such as whether this stratification occurs in mixed species biofilms and the relevance to *in vivo* infections, the ability to distinguish different forms of Psl within mature biofilms highlights the complexity of these structures.

To our knowledge, differential staining of a single polysaccharide within a biofilm has not been observed, which is likely due to a lack of appropriate reagents. We have performed a variety of experiments to confirm our differential staining results, including: 1) evaluating staining against multiple *P*. *aeruginosa* isolates, 2) staining of live or fixed (e.g. acetone vs. 4% paraformaldehyde) biofilms, 3) altering the order of antibody staining and washing steps, or 4) interchanging fluorophores associated with each mAb, or 5) evaluating binding against strains mutated for expression of other polysaccharides (e.g. Pel) or that alter Psl expression levels (e.g. *wspF*, *cdrA*, *pelF* or *pslG*) (Unpublished observations, V.A.R and D.J.W.). In all of our analyses, Psl epitope stratification is conserved.

Many studies in recent years have examined the role of both polyclonal antibodies and mAbs to disrupt biofilms. For example, similar to what we have shown here, polyclonal antibodies against the PhnD protein of *Staphylococcal* species can block attachment and aggregation, as well as promote biofilm disruption in the presence of neutrophils^42^. Additionally, mAbs against the Aap protein of *Staphylococcus epidermidis* can inhibit biofilm formation^43,44^. Another promising example is mAbs directed against the DNABII family of proteins, which is found ubiquitously in both Gram-positive and Gram-negative bacteria. Studies utilizing various mAbs to these proteins have demonstrated that they not only disrupt biofilms of various species^45,46^, but can cause release of bacteria, which are then more susceptible to antibiotic treatment^46^.

While development of new small molecule antibiotics is also important in contributing to the fight against antimicrobial resistance, pathogen-specific mAbs have several advantages. The use of broad-spectrum antibiotics is a driver of resistance in both targeted and non-targeted microorganisms. These agents also alter the microbiota, which can reduce bacterial competition against problem pathogens. In contrast, mAbs are directed against a single pathogen or virulence mechanism, thus use of these agents would not lead to collateral damage of the beneficial microbiome, which is a common side effect of broad-spectrum agents. In addition, unlike antibiotics, mAbs exert less pressure on resistance since they do not directly kill bacteria. Furthermore, mAbs naturally have long half-lives (up to 4 weeks), which can be engineered to offer extended coverage (up to 3 months)^47^. Moreover, given the low metabolic activity of bacterial cells within biofilms, antibiotics with mechanisms of action requiring cell division are simply not effective in this context^48–50^. However, approaches that can disrupt biofilm communities in combination with adjunctive antibiotic use, such as the work described herein, holds significant promise in mitigating biofilm infections or in preventing spread of bacteria to distal sites.

In conclusion, we demonstrate that anti-Psl mAbs are capable of inhibiting early biofilm events and promote bacterial clearance from preformed biofilms in the presence of host-effector cells. Under these latter assay conditions, anti-Psl mAbs were more effective than antibiotics in promoting clearance, however adjunctive use of both mAbs and antibiotics yielded enhanced biomass reduction than either drug alone. Interestingly, we also show that the class I, II and III Psl epitopes stain distinct locations within mature biofilms, which correlated with the ability of neutrophils to access and kill bacteria. Overall, our data suggests that anti-Psl mAbs are promising candidates for treatment of established or persistent *P*. *aeruginosa* infections.

## Materials and Methods

### Bacterial strains and media

*Pseudomonas aeruginosa* strain PAO1 was the primary strain used in this study^51^. Strains CF127, MSH3, ARC3928 were previously described^36,52^. Bacterial strains were grown aerobically at 37 °C in Luria Broth without sodium chloride (LBNS) or Jensen’s medium^53^; LBNS agar plates were solidified with 1.5% agar (Fisher Scientific). When necessary, arabinose (Sigma-Aldrich) was added to a final concentration of 0.5%.

### Chronic porcine wound model

All procedures were performed in accordance with federal, state, and institutional guidelines and were approved by the Ohio State University Institutional Animal Care and Use Committee (IACUC). Briefly, female domestic swine were anesthetized and subjected to thermal injury on their dorsal trunk and, three days later, inoculated with *P*. *aeruginosa* strain PAO1, as previously described^6^. At day 14 post-infection, biopsies were collected and sectioned, as per^54^. Sections were then washed in 1xPBS, blocked with 1% BSA (Sigma-Aldrich) for 30 min. at room temperature (RT), and stained with Alexa Fluor labeled anti-Psl antibodies (10 µg/ml) in 1% BSA for 1 h. at RT, then moved to 4 °C overnight. Lastly, sections were washed in 1xPBS and incubated with DAPI (200 µg/ml; Thermo Fisher Scientific) in 1xPBS. Samples were rinsed in ddH2O, mounted with ProLong Gold Antifade (Molecular Probes), and imaged at the Ohio State University Campus Microscopy and Imaging Facility on an Olympus Fluoview 1000 laser scanning confocal microscope. Images were obtained with a 10 × objective and processed using the Olympus FV1000 Viewer.

### Biofilm attachment assay

Overnight cultures were diluted (50 µl) into fresh LBNS, grown, and normalized to an OD_600_ = 0.5. Cultures were centrifuged at 20,000 × g for 3 min and the pellet re-suspended in 1 ml LBNS. Psl mAbs (10 µg/ml) were added to the bacterial suspension and 100 µl was aliquoted into a polyvinyl microtiter dish (Corning). After 2 hours of incubation at room temperature, the wells were washed in H_2_O and 0.1% crystal violet was added for 30 minutes. Plates were washed in H_2_O and the remaining crystal violet solubilized in 95% ethanol, transferred to a polycarbonate microtiter plate (Corning), and read at 540 nm. Data was analyzed and graphed in GraphPad Prism with results presented as percent inhibition of Psl-mediated attachment, such that any attachment mediated by the negative control strain was subtracted from the data before setting the IgG control to 100%. Raw data is provided in Supplementary Table S1.

### Planktonic aggregation assay

Cultures of PAO1 P_BAD_-*psl* were grown in Jensen’s medium overnight and diluted into fresh Jensen’s medium with or without arabinose (0.5%). Psl mAbs were added individually (20 µg/ml) or in combination (6.7 µg/ml ea.) and the cultures incubated for 2 h at 37 °C. Congo Red (Sigma-Aldrich) was added (40 µg/ml) and tubes were placed back in incubation for the final 15 min. Culture density was measured from 1 ml samples at an OD_600_. Another 1 ml sample was removed and centrifuged (20,000 × g for 10 min.) and the A_490_ was measured to determine the amount of Congo Red remaining in solution. An index was calculated (A_490_/A_600_) and the data graphed and analyzed via GraphPad Prism with results presented as percent inhibition of Psl-mediated aggregation, such that any aggregation mediated by the negative control strain was subtracted from the data before setting the IgG control to 100%. Raw data is provided in Supplementary Table S2.

### Neutrophil isolation

Human neutrophils were obtained, with informed consent, from healthy adult donors using an approved IRB protocol (2009H0314) at The Ohio State University. While following all relevant guidelines and regulations, neutrophils were isolated as previously described^26^ and kept on ice prior to use.

### Oxidative burst assay

The luminol chemiluminescence assay was used to detect intracellular and extracellular reactive oxygen species (ROS) generated by neutrophils upon interaction with the bacteria in the presence of the anti-Psl mAbs (Engels *et al*., 1985). Polymyristic acid (PMA) was used at a concentration of 10 ng/ml as a positive control for the generation of ROS. Briefly, neutrophils were diluted to a final concentration of 4 × 10^6^/ml in HBSS containing 50 mM luminol and seeded onto a 96-well microtiter plate. A mixture of bacteria and the ant-Psl mAbs (10 µg/ml; individually or in combination) was added to the neutrophils. The ROS generation was measured at regular intervals over one hour by luminescence using a SpectraMax M5 plate reader (Molecular Devices LLC, Sunnyvale, CA). Relative light units (RLU) were plotted as a function of time to evaluate chemiluminescence (CL) rate^55^.

### Flow cell biofilm formation

Overnight cultures in LBNS were diluted into fresh LBNS and grown to an OD_600_ = 0.5–0.7. Cultures (200 µl) were seeded into ibidi µ-Slide VI^0.4^ flow cells (ibidi) and incubated at 37 °C for 2 h to allow for cell attachment. Flow was then initiated (0.4 ml/min) using 5% LBNS and maintained for 18 h, after which point the flow cells were washed 2X with 1XPBS and either subjected to treatment (see biofilm disruption assay below) or fixed using 4% paraformaldehyde (Affymetrix).

### Static biofilm formation

Overnight cultures in LBNS were diluted to an OD_600_ = 0.5 in fresh LBNS and 100 µl was spotted onto the glass coverslip in a 35mm MatTeck glass bottom plate (Part # P35G-1.5-10-C). Plates were incubated in a humidified chamber at room temperature for 48 h, after which point they were washed 2x with 1XPBS and fixed using 4% paraformaldehyde (Affymetrix).

### Biofilm staining with anti-Psl mAbs

After fixation, samples were washed in 1xPBS and incubated with anti-Psl mAbs (10 µg/ml) in 1xPBS for 1 h at RT. Samples were washed in 1xPBS and remained in 1xPBS for CLSM. All confocal images were taken at the Ohio State University Campus Microscopy and Imaging Facility on an Olympus Fluoview 1000 laser scanning confocal microscope or Nikon A1Rsi Resonant scanning inverted confocal system. Images were obtained with either a 10x or 100x objective, as indicated, and processed using IMARIS.

### Biofilm disruption assay

Flow cell biofilms (18 h) were prepared as described above. Isolated neutrophils were stained with Cell Tracker Blue (Molecular Probes) for 1 h at 4 °C, washed 3X with RPMI medium (Corning) to remove excess stain, and suspended in 1 ml of RPMI. IgG control (20 µg/ml) or anti-Psl mAbs alone (20 µg/ml) or in combination (6.7 µg/ml) were mixed with neutrophils, either in the absence or presence of meropenem (0.5, 3, or 30 µg/ml), and passed over flow cell grown PAO1 GFP expressing biofilms 5X (180 µl each time), leaving the solution on the biofilm at the final pass. Flow cells were then incubated at 37 °C with 5% CO_2_ for 4 hours, after which point the chambers were washed 3X with 1XPBS and fixed for 30 minutes using 4% paraformaldehyde. After fixation, the chambers were washed 3X with 1XPBS and imaged via CLSM. Data were quantified using IMARIS, compiled, and the standard error of the mean (SEM) determined.

### Statistical analysis

Individual comparison was analyzed by student’s t-test. IN some experiments, the area under the curve (AUC) was estimated using the Trapezoidal rule. A one-way ANOVA model with heterogeneous within group variance was applied and Dunnett’s test was used for comparisons to IgG controls and multiplicity adjusted p-values. Prism (GraphPad) or SAS 9.3 (SAS Institute) was used for statistical analyses.

## Electronic supplementary material

Supplemental Material

Supplemental Video
