# Publisher Correction: Anti-Psl Targeting of *Pseudomonas aeruginosa* Biofilms for Neutrophil-Mediated Disruption

**DOI:** 10.1038/s41598-018-26660-6

**Published:** 2018-06-20

**Authors:** Valerie A. Ray, Preston J. Hill, C. Kendall Stover, Sashwati Roy, Chandan K. Sen, Li Yu, Daniel J. Wozniak, Antonio DiGiandomenico

**Affiliations:** 1 Center for Microbial Interface Biology, Departments of Microbial Infection and Immunity, Microbiology, Ohio State University, Columbus, OH 43210 USA; 2 Department of Infectious Diseases, MedImmune, LLC, Gaithersburg, MD 20878 USA; 3 Comprehensive Wound Center, Davis Heart and Lung Research Institute, Center for Regenerative Medicine and Cell Based Therapies, Ohio State University Medical Center, Columbus, OH 43210 USA; 4 Department of Surgery, College of Veterinary Medicine, Ohio State University, Columbus, OH 43210 USA; 5 Translational Sciences, MedImmune, LLC, Gaithersburg, MD 20878 USA

Correction to: *Scientific Reports* 10.1038/s41598-017-16215-6, published online 22 November 2017

The original version of this Article contained an error in the spelling of the author C. Kendall Stover, which was incorrectly given as Kendall C. Stover.

This error has now been corrected in the PDF and HTML versions of the paper.

