# Supplementary material for: Anti-Psl Targeting of Pseudomonas aeruginosa Biofilms for Neutrophil-Mediated Disruption
Source: Sci Rep. 2017 Nov 22;7:16065. doi: 10.1038/s41598-017-16215-6 (PMC5700069; doi:10.1038/s41598-017-16215-6)
Supplement: Supplementary file 1 — Supplemental Material [file 41598_2017_16215_MOESM1_ESM.pdf]

# Anti-Psl Targeting of *Pseudomonas aeruginosa* Biofilms for Neutrophil-Mediated Disruption

Valerie A. Ray[1], Preston J. Hill[1], C. Kendall Stover[2], Sashwati Roy[3,4], Chandan K. Sen[3,4], Li Yu[5], Daniel J. Wozniak[1], Antonio DiGiandomenico[2*]

[1] Center for Microbial Interface Biology, Departments of Microbial Infection and Immunity, Microbiology, Ohio State University, Columbus, OH 43210, USA.

[2] Department of Infectious Diseases, MedImmune, LLC, Gaithersburg, MD 20878, USA.

[3] Comprehensive Wound Center, Davis Heart and Lung Research Institute, Centers for Regenerative Medicine and Cell Based Therapies, Ohio State University Medical Center, Columbus, OH 43210, USA.

[4] Department of Surgery, College of Veterinary Medicine, Ohio State University, Columbus, OH 43210, USA.

[5] Translational Sciences, MedImmune, LLC, Gaithersburg, MD 20878, USA.

**Supplemental Information**

**Supplementary Fig. S1. Structure of Psl and synthetic Psl oligosaccharides.** (A) Cell-free Psl is a pentasaccharide repeat unit. (B-E) Synthetic Psl oligosaccharides used to evaluate binding of anti-Psl mAbs which recognized unique epitopes (class I, II and III) [1]. (B) Psl tetrasaccharide – bound by the class II mAb. (C) Psl pentasaccharide – bound by the class II mAb, (D) Psl hexasaccharide – bound by class II and class III mAbs. (E) Psl di-pentasaccharide – bound by the class II mAb and weakly by the class III mAb.

**Supplementary Fig. S2.** Individual anti-Psl mAbs stain PAO1 biofilms. Flow grown biofilms of PAO1 stained with either (A) class I (green), (B) class II (orange), or (C) class III (red) anti-Psl mAbs, imaged via CLSM at 10x magnification, and processed via IMARIS. Scale bars represent 150 µm.

**Supplementary Fig. S3. Thermally injured porcine tissue does not react with an isotype control IgG mAb.** *P. aeruginosa* infected skin from thermally injured pigs was imaged via CLSM at 10x magnification. (A) DIC image (same as Fig. 3), (B) DAPI staining, and (C) control IgG mAb staining. (D-F) The wound bed is below the white dotted line and white dashed line represents below the wound bed. Scale bars represent 100 µm.

**Supplementary Figure S4. Anti-Psl mAbs elicit a phagocytic burst response from human neutrophils**. Planktonic PAO1 was incubated with neutrophils and PMA (positive control), non-opsonized or IgG isotype control antibody (negative controls), or

opsonized with human serum, individual anti-Psl mAbs, or a combination of all three mAbs.

**Supplementary Movie S1. Anti-Psl mAbs differentially stain the biofilm.** IMARIS generated movie from image shown in Figure 2B with removal of layers (class I – green, class II – yellow/orange, and class III – red) and rotation to demonstrate differential staining.

**Supplementary Fig. S1**

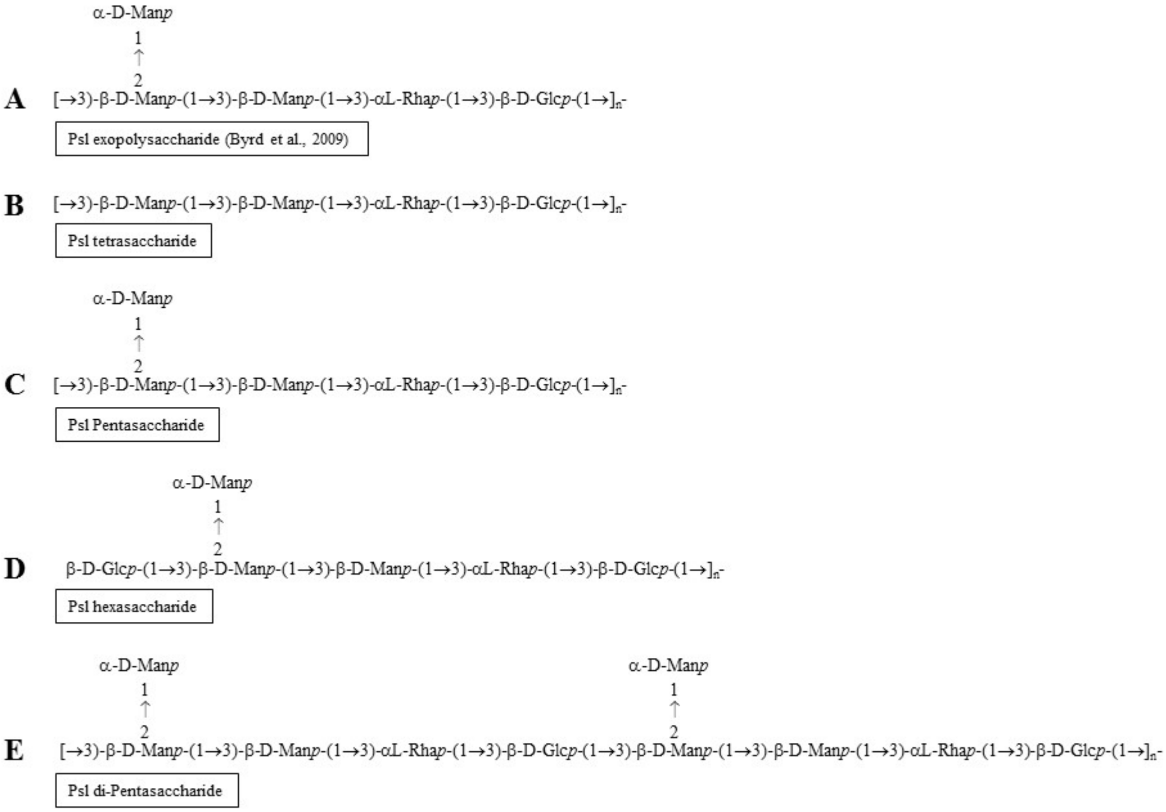

**Supplementary Fig. 2**

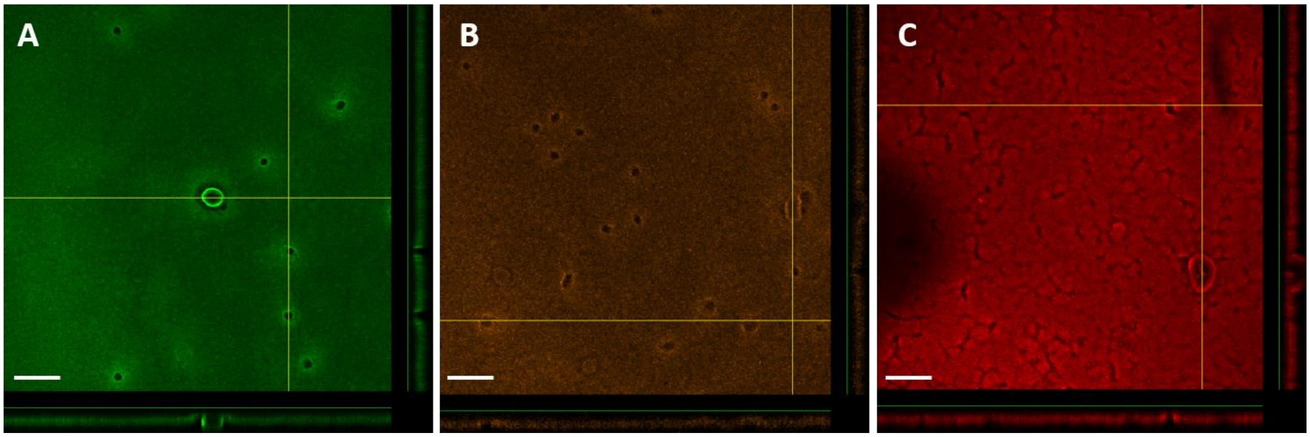

**Supplementary Fig. S3**

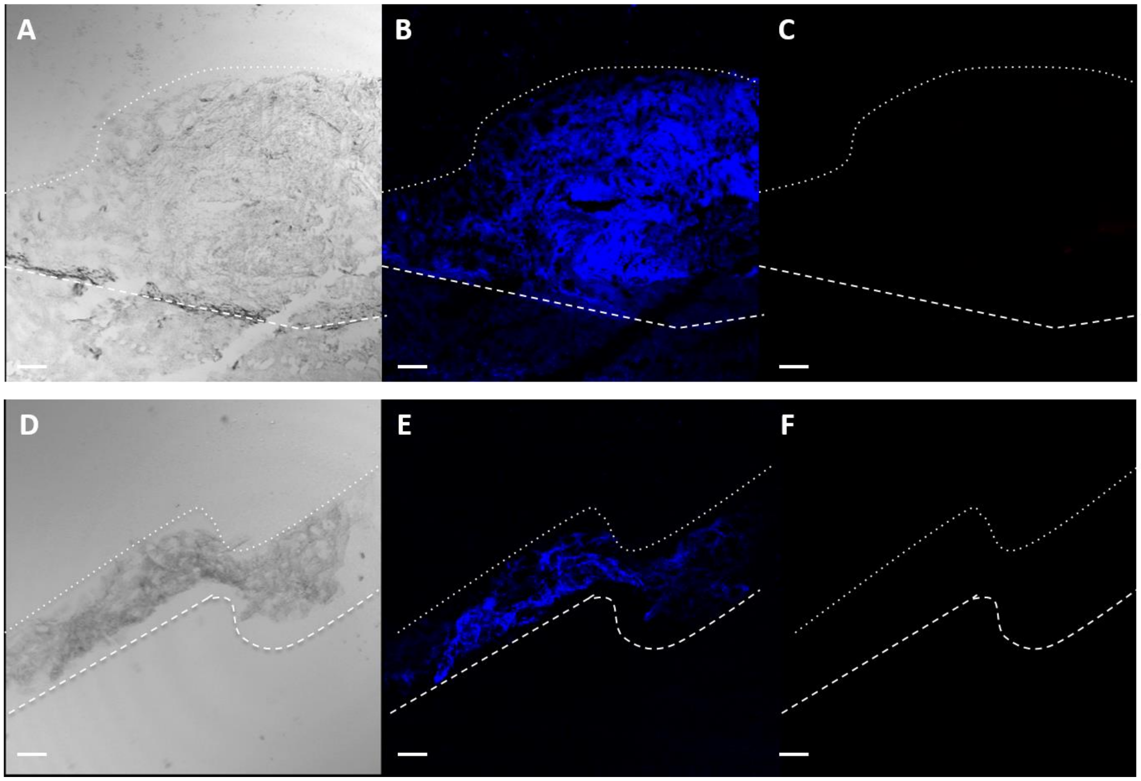

**Supplementary Fig. S4**

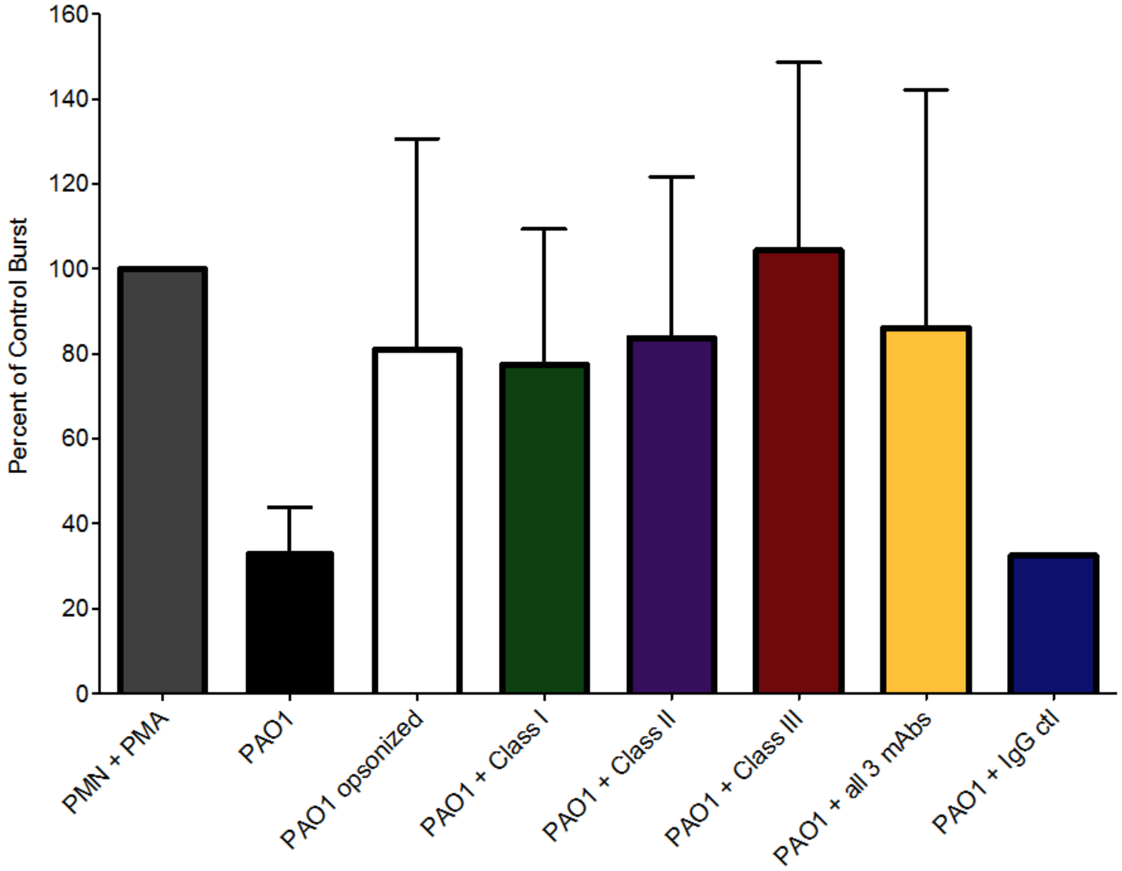

**Supplementary TABLE S1.** Raw data from biofilm attachment assays.

*Experiment 1*

| Condition | O.D. $A_{540}$ | Condition | O.D. $A_{540}$ |
|---|---|---|---|
| Δ*psl* #1 | 0.033 | PAO1 #1 | 0.121 |
| Δ*psl* #2 | 0.038 | PAO1 #2 | 0.158 |
| Δ*psl* #3 | 0.050 | PAO1 #3 | 0.128 |
| IgG Control #1 | 0.111 | Class I #1 | 0.037 |
| IgG Control #2 | 0.150 | Class I #2 | 0.056 |
| IgG Control #3 | 0.135 | Class I #3 | 0.070 |
| Class II #1 | 0.070 | Class III #1 | 0.080 |
| Class II #2 | 0.126 | Class III #2 | 0.081 |
| Class II #3 | 0.141 | Class III #3 | 0.059 |
| All 3 mAbs #1 | 0.102 | | |
| All 3 mAbs #2 | 0.121 | | |
| All 3 mAbs #3 | 0.106 | | |

*Experiment 2*

| Condition | O.D. $A_{540}$ | Condition | O.D. $A_{540}$ |
|---|---|---|---|
| Δ*psl* #1 | 0.049 | PAO1 #1 | 0.146 |
| Δ*psl* #2 | 0.060 | PAO1 #2 | 0.155 |
| Δ*psl* #3 | 0.057 | PAO1 #3 | 0.166 |
| IgG Control #1 | 0.143 | Class I #1 | 0.066 |
| IgG Control #2 | 0.160 | Class I #2 | 0.129 |
| IgG Control #3 | 0.150 | Class I #3 | 0.116 |
| Class II #1 | 0.128 | Class III #1 | 0.162 |
| Class II #2 | 0.112 | Class III #2 | 0.088 |
| Class II #3 | 0.137 | Class III #3 | 0.071 |
| All 3 mAbs #1 | 0.122 | | |
| All 3 mAbs #2 | 0.134 | | |
| All 3 mAbs #3 | 0.105 | | |

*Experiment 3*

| Condition | O.D. $A_{540}$ | Condition | O.D. $A_{540}$ |
|---|---|---|---|
| Δ*psl* #1 | 0.031 | PAO1 #1 | 0.155 |
| Δ*psl* #2 | 0.059 | PAO1 #2 | 0.174 |
| Δ*psl* #3 | 0.061 | PAO1 #3 | 0.168 |
| IgG Control #1 | 0.164 | Class I #1 | 0.080 |
| IgG Control #2 | 0.173 | Class I #2 | 0.122 |
| IgG Control #3 | 0.159 | Class I #3 | 0.047 |

| | | | |
|---|---|---|---|
| Class II #1 | 0.293 | Class III #1 | 0.155 |
| Class II #2 | 0.108 | Class III #2 | 0.107 |
| Class II #3 | 0.115 | Class III #3 | 0.102 |
| | | | |
| All 3 mAbs #1 | 0.104 | | |
| All 3 mAbs #2 | 0.092 | | |
| All 3 mAbs #3 | 0.104 | | |

*Experiment 4*

| Condition | O.D. $A_{540}$ | Condition | O.D. $A_{540}$ |
|---|---|---|---|
| Δ*psl* #1 | 0.067 | PAO1 #1 | 0.129 |
| Δ*psl* #2 | 0.067 | PAO1 #2 | 0.152 |
| Δ*psl* #3 | 0.048 | PAO1 #3 | 0.147 |
| | | | |
| IgG Control #1 | 0.105 | Class I #1 | 0.103 |
| IgG Control #2 | 0.125 | Class I #2 | 0.117 |
| IgG Control #3 | 0.155 | Class I #3 | 0.127 |
| | | | |
| Class II #1 | 0.111 | Class III #1 | 0.110 |
| Class II #2 | 0.122 | Class III #2 | 0.112 |
| Class II #3 | 0.133 | Class III #3 | 0.133 |
| | | | |
| All 3 mAbs #1 | 0.106 | | |
| All 3 mAbs #2 | 0.121 | | |
| All 3 mAbs #3 | 0.120 | | |

**Supplementary TABLE S2.** Raw data from biofilm aggregation assays.

*Experiment 1*

| Condition | O.D. $A_{600}$ | O.D.$A_{490}$ | Aggregation Index ($A_{490}$/$A_{600}$) |
|---|---|---|---|
| Δ*psl* #1 | 0.091 | 1.134 | 12.462 |
| Δ*psl* #2 | 0.093 | 1.122 | 12.065 |
| Δ*psl* #3 | 0.093 | 1.142 | 12.280 |
| WFPA 801 #1 | 0.072 | 1.196 | 16.611 |
| WFPA 801 #2 | 0.071 | 1.114 | 15.690 |
| WFPA 801 #3 | 0.074 | 1.160 | 15.676 |
| WFPA 801 + ara #1 | 0.040 | 0.940 | 23.500 |
| WFPA 801 + ara #2 | 0.043 | 1.000 | 23.256 |
| WFPA 801 + ara #3 | 0.045 | 1.044 | 23.200 |
| IgG Control #1 | 0.044 | 1.000 | 22.727 |
| IgG Control #2 | 0.042 | 0.982 | 23.381 |
| IgG Control #3 | 0.044 | 1.018 | 23.136 |
| Class I #1 | 0.054 | 1.068 | 19.778 |
| Class I #2 | 0.053 | 1.036 | 19.547 |
| Class I #3 | 0.056 | 1.092 | 19.500 |
| Class II #1 | 0.053 | 1.066 | 20.113 |
| Class II #2 | 0.055 | 1.058 | 19.236 |
| Class II #3 | 0.048 | 1.038 | 21.625 |
| Class III #1 | 0.049 | 1.100 | 22.449 |
| Class III #2 | 0.048 | 1.068 | 22.208 |
| Class III #3 | 0.050 | 1.114 | 22.280 |
| All 3 mAbs #1 | 0.066 | 1.210 | 18.333 |
| All 3 mAbs #2 | 0.059 | 1.068 | 18.102 |
| All 3 mAbs #3 | 0.060 | 1.136 | 18.933 |

*Experiment 2*

| Condition | O.D. $A_{600}$ | O.D.$A_{490}$ | Aggregation Index ($A_{490}$/$A_{600}$) |
|---|---|---|---|
| Δ*psl* #1 | 0.090 | 1.016 | 11.289 |
| Δ*psl* #2 | 0.094 | 1.111 | 11.819 |
| Δ*psl* #3 | 0.103 | 1.158 | 11.243 |
| WFPA 801 #1 | 0.071 | 1.098 | 15.465 |
| WFPA 801 #2 | 0.074 | 1.128 | 15.243 |
| WFPA 801 #3 | 0.080 | 1.210 | 15.125 |
| WFPA 801 + ara #1 | 0.053 | 1.106 | 20.868 |
| WFPA 801 + ara #2 | 0.055 | 1.130 | 20.545 |
| WFPA 801 + ara #3 | 0.059 | 1.186 | 20.102 |

| | | | |
|---|---|---|---|
| IgG Control #1 | 0.056 | 1.112 | 19.857 |
| IgG Control #2 | 0.059 | 1.100 | 18.644 |
| IgG Control #3 | 0.057 | 1.168 | 20.491 |
| Class I #1 | 0.057 | 1.054 | 18.491 |
| Class I #2 | 0.057 | 1.096 | 19.228 |
| Class I #3 | 0.059 | 1.102 | 18.678 |
| Class II #1 | 0.056 | 1.044 | 18.643 |
| Class II #2 | 0.058 | 1.138 | 19.621 |
| Class II #3 | 0.058 | 1.162 | 20.034 |
| Class III #1 | 0.051 | 1.038 | 20.353 |
| Class III #2 | 0.058 | 1.344 | 23.172 |
| Class III #3 | 0.057 | 1.122 | 19.684 |
| All 3 mAbs #1 | 0.062 | 0.994 | 16.032 |
| All 3 mAbs #2 | 0.063 | 1.008 | 16.000 |
| All 3 mAbs #3 | 0.066 | 1.060 | 16.061 |

*Experiment 3*

| Condition | O.D. $A_{600}$ | O.D.$A_{490}$ | Aggregation Index ($A_{490}/A_{600}$) |
|---|---|---|---|
| Δ*psl* #1 | 0.120 | 0.849 | 7.075 |
| Δ*psl* #2 | 0.110 | 0.877 | 7.973 |
| Δ*psl* #3 | 0.122 | 0.816 | 6.689 |
| WFPA 801 #1 | 0.107 | 1.076 | 10.056 |
| WFPA 801 #2 | 0.112 | 1.096 | 9.786 |
| WFPA 801 #3 | 0.126 | 1.124 | 8.921 |
| WFPA 801 + ara #1 | 0.084 | 1.050 | 12.500 |
| WFPA 801 + ara #2 | 0.086 | 1.146 | 13.326 |
| WFPA 801 + ara #3 | 0.090 | 1.158 | 12.867 |
| IgG Control #1 | 0.088 | 0.906 | 10.295 |
| IgG Control #2 | 0.089 | 0.988 | 11.101 |
| IgG Control #3 | 0.081 | 0.892 | 11.012 |
| Class I #1 | 0.097 | 1.064 | 10.969 |
| Class I #2 | 0.089 | 1.030 | 11.573 |
| Class I #3 | 0.096 | 1.132 | 11.792 |
| Class II #1 | 0.082 | 0.986 | 12.024 |
| Class II #2 | 0.085 | 1.014 | 11.929 |
| Class II #3 | 0.090 | 1.024 | 11.378 |
| Class III #1 | 0.086 | 1.032 | 12.000 |
| Class III #2 | 0.085 | 1.028 | 12.094 |
| Class III #3 | 0.084 | 1.018 | 12.119 |
| All 3 mAbs #1 | 0.081 | 0.992 | 12.247 |

| | | | |
|---|---|---|---|
| All 3 mAbs #2 | 0.082 | 0.980 | 11.951 |
| All 3 mAbs #3 | 0.087 | 1.032 | 11.862 |

*Experiment 4*

| Condition | O.D. $A_{600}$ | O.D.$A_{490}$ | Aggregation Index ($A_{490}$/$A_{600}$) |
|---|---|---|---|
| Δ*psl* #1 | 0.198 | 0.973 | 4.914 |
| Δ*psl* #2 | 0.209 | 0.903 | 4.321 |
| Δ*psl* #3 | 0.208 | 0.991 | 4.764 |
| WFPA 801 #1 | 0.213 | 1.078 | 5.061 |
| WFPA 801 #2 | 0.215 | 1.184 | 5.507 |
| WFPA 801 #3 | 0.249 | 1.166 | 4.683 |
| WFPA 801 + ara #1 | 0.104 | 1.002 | 9.635 |
| WFPA 801 + ara #2 | 0.106 | 0.980 | 9.245 |
| WFPA 801 + ara #3 | 0.098 | 1.042 | 10.633 |
| IgG Control #1 | 0.109 | 1.022 | 9.376 |
| IgG Control #2 | 0.101 | 1.018 | 10.079 |
| IgG Control #3 | 0.105 | 1.058 | 10.076 |
| Class I #1 | 0.132 | 0.894 | 6.773 |
| Class I #2 | 0.124 | 0.838 | 6.758 |
| Class I #3 | 0.134 | 0.928 | 6.925 |
| Class II #1 | 0.132 | 0.950 | 7.197 |
| Class II #2 | 0.142 | 0.966 | 6.803 |
| Class II #3 | 0.138 | 1.114 | 8.072 |
| Class III #1 | 0.140 | 1.098 | 7.843 |
| Class III #2 | 0.133 | 1.050 | 7.895 |
| Class III #3 | 0.130 | 1.062 | 8.169 |
| All 3 mAbs #1 | 0.153 | 1.006 | 6.575 |
| All 3 mAbs #2 | 0.161 | 1.106 | 6.870 |
| All 3 mAbs #3 | 0.164 | 1.080 | 6.585 |

## References

1 Li, H. *et al.* Epitope mapping of monoclonal antibodies using synthetic oligosaccharides uncovers novel aspects of immune recognition of the Psl exopolysaccharide of Pseudomonas aeruginosa. *Chemistry* **19**, 17425-17431, doi:10.1002/chem.201302916 (2013).
